# Transforaminal Decompression and Interbody Fusion in the Treatment of Thoracolumbar Fracture and Dislocation with Spinal Cord Injury

**DOI:** 10.1371/journal.pone.0105625

**Published:** 2014-08-22

**Authors:** Ai-Min Wu, Yi-Jing Zheng, Yan Lin, Yao-Sen Wu, Fang-Min Mao, Wen-Fei Ni, Xiang-Yang Wang, Hua-Zi Xu

**Affiliations:** Department of Orthopedics, Second Affiliated Hospital of Wenzhou Medical University, Zhejiang Spinal Research Center, Wenzhou, Zhejiang, People's Republic of China; University of Michigan, United States of America

## Abstract

**Study Design:**

A retrospective clinical study.

**Objective:**

To evaluate the efficacy and safety of transforaminal decompression and interbody fusion in the treatment of thoracolumbar fracture and dislocation with spinal cord injury.

**Methods:**

Twenty-six spinal cord injured patients with thoracolumbar fracture and dislocation were treated by transforaminal decompression and interbody fusion. The operation time, intraoperative blood loss, and complications were recorded; the Cobb angle and compressive rate (CR) of the anterior height of two adjacent vertebrae were measured; and the nerve injury was assessed according to sensory scores and motor scores of the American Spinal Injury Association (ASIA) standards for neurological classification of spinal cord injury.

**Results:**

The operative time was 250±57 min, and intraoperative blood loss was 440±168 ml. Cerebrospinal leakage was detected and repaired during the operation in two patients. A total of 24 of 26 patients were followed up for more than 2 years. ASIA sensory scores and motor scores were improved significantly at 3 months and 6 months after operation; the Cobb angle and CR of the anterior height of two adjacent vertebrae were corrected and showed a significant difference at post-operation; and the values were maintained at 3 months after operation and the last follow-up.

**Conclusion:**

We showed that transforaminal decompression together with interbody fusion is an alternative method to treat thoracolumbar fracture and dislocation.

## Introduction

Thoracolumbar fractures and dislocations with spinal cord injury, especially those with spinal cord compression due to a damaged anterior intervertebral disc and bony fragments, lead to nerve injury. Anterior decompression or combined anterior and posterior decompression and fusion are recommended by surgeons [1], [2], [3].

Although the anterior approach can remove the damaged intervertebral disc and bony fragments directly, the complications and risks of anterior vascular and nerve injury are considerable [4], [5]. Therefore, surgeons are always seeking better techniques that are more minimally invasive, simpler to perform, less risky, and can reach the same efficacy as traditional surgery.

Transforaminal lumbar interbody fusion (TLIF) has been reported as a treatment for lumbar disc herniation and lumbar stenosis [6], [7], [8]. The TLIF technique can remove the anterior compressed intervertebral disc and bony fragments, avoiding the decompression procedure of the anterior approach. In 2011, Fang *et al*. [9] used the TLIF technique to treat patient with an old T12/L1 fracture and dislocation. Compared to the posterior lumbar interbody fusion technique, transforaminal interbody fusion can be used to treat a fracture and dislocation above the lumbar spine [7], [9], [10]. Here, we retrospectively reviewed 24/26 consecutive patients (two patients were lost) with thoracolumbar fractures and dislocations treated by transforaminal decompression and interbody fusion.

## Materials and Methods

### Patient population

From June 2007 to August 2011, a total of 26 consecutive patients with thoracolumbar fractures and dislocations treated by transforaminal decompression and interbody fusion in our department were enrolled in this study. There were 17 males and 9 females, with an average age of 32.5 years old (range: 21–54 years old). The diagnosis of a thoracolumbar fracture and dislocation was confirmed by both plain radiography and two-dimensional reconstructive computed tomography (CT) imaging. Magnetic resonance imaging (MRI) was obtained preoperatively to assess the spinal cord and interspinous or posterior longitudinal ligament injury. The involved discs were as follows: T9/T10 (1 case), T11/T12 (8 cases), T12/L1 (10 cases), L1/L2 (5 cases), L2/3 (2 cases). All cases were fractures of type C, according to the latest AOSpine Thoracolumbar Spine Injury Classification System [11].

### Inclusion criteria

The inclusion criteria were as follows: 1) fracture and dislocation at the thoracolumbar region; 2) spinal cord injury with motor and sensory deficiency; 3) the damage was limited to the intervertebral disc and the adjacent endplate; and 4) single segmental thoracolumbar fracture and dislocation.

### Exclusion criteria

The exclusion criteria were as follows: 1) thoracolumbar fracture without dislocation; 2) severe dislocation in which the whole upper vertebra was dislocated at the front of the lower vertebra; 3) severe vertebral body fracture in which titanium mesh was recommended to reconstruct the spine sequence; 4) multi-segmental thoracolumbar fracture and dislocation.

### Images and nerve injury assessment

**Figure 1:**
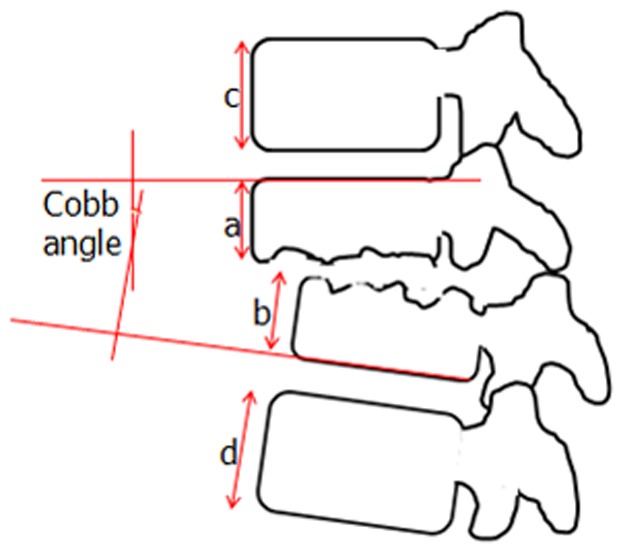
The measurements of Cobb angle and anterior height. The  = compressive rate (CR) of the anterior height was calculated by the formula: CR%  = 100%−(a+b)/(c+d)×100%.



The Cobb angle and compressive rate (CR) of the anterior height of two adjacent vertebrae were measured preoperatively, postoperatively, 3 months after theoperation, and at the last follow-up and are shown in **Figure 1**. The CR of the anterior height was calculated by the formula:

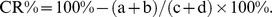



The nerve injury was assessed according to sensory scores and motor scores of the American Spinal Injury Association (ASIA) standards for neurological classification of spinal cord injury [12] at the following time points: pre-operation, 3 months after operation, 6 months after operation, 12 months after operation, and the last follow-up.

### Operation procedure

The patient was placed on the operating table in the prone position. After general anesthesia and target segment was located by C-arm X-ray, a midline incision was made and the paraspinal muscle was separated from the lamina bilaterally.

Suitable pedicle screws were inserted into the upper and lower vertebrae or second adjacent vertebrae, and the left or right facet joint was resected. After hemostasis of the epidural and radicular veins by bipolar forceps, the intervertebral disc was exposed after protection of the nerve root and thecal sac, the damaged disc was removed, the upper and lower cortical endplates were removed with straight and curved osteotomes until the cortical endplate was stripped, and the best conditions for the bony fusion were achieved. The height of the intervertebral space was measured by a tryout cage, and then a correct-sized bone from the ilium or interbody cage packed with granulated autogenous bone was inserted into the intervertebral space.

Next, the rods were installed, to restore the height and realign the spinal sequence, and the position of the internal instruments was confirmed by C-arm X-ray. Finally, a drain was placed and the wound was checked for hemostasis and closure. The operative time and intraoperative blood loss were recorded by the anesthetists, and the data were extracted from the anesthesia record sheet.

### Ethics consideration

This research was performed following the principles described in the Declaration of Helsinki and was approved by the Institutional Ethics Review Board of our hospital. Written informed consent was obtained from all participants.

### Statistical analysis

The data were analyzed with standard software (SPSS, version 17.0, SPSS Inc., Chicago, IL, USA). The preoperative, postoperative (immediately, 3 months, and 6 months after operation), and final data (more than 2 years after operation, averaged 34.9 months) were tested by repeated measures analysis of variance (ANOVA). The level of significance was set at P<0.05.

## Results

**Figure 2:**
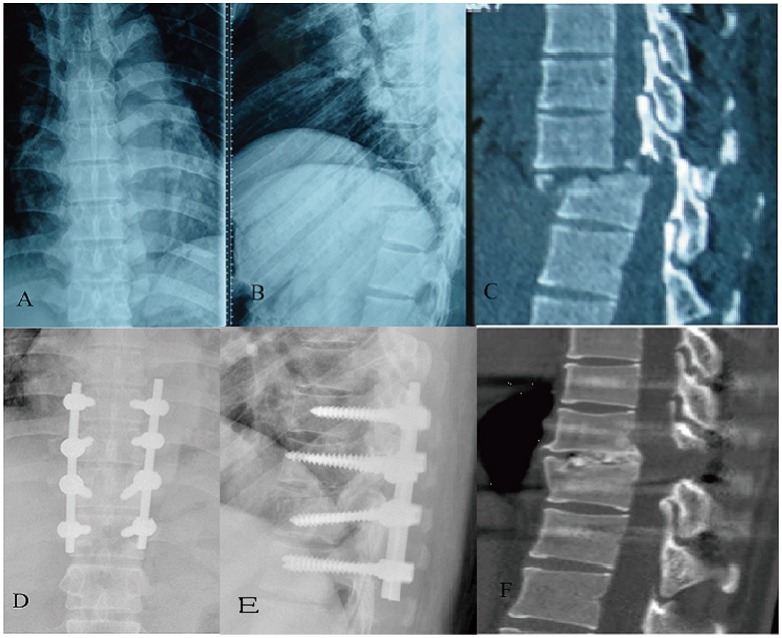
The anteroposterior and lateral X-ray films (A and B) of a 34-year-old man sent to our hospital after an accidental fall show a T9–T10 fracture and dislocation, and the injury was confirmed by CT reconstruction (C). The patient's ASIA sensory score was 173, and his motor score was 67. We performed transforaminal decompression and interbody fusion on him. The anteroposterior and lateral X-ray films (D and E) at 3 months postoperation show the internal instruments placed in a good position. Intervertebral bony fusion between T9–T10 was observed on the CT images at 36 months postoperation (F). The patient's ASIA sensory score was 216, and his motor score was 96.

The operative time was 250±57 min, and intraoperative blood loss was 440±168 ml. Cerebrospinal leakage was detected and repaired during the operation in two patients. A total of 24 of 26 patients were followed up for more than 2 years (one male and one female were lost and could not be contacted), for an average of 34.9 months (range, 24–63 months). Intervertebral bony fusion was achieved in all patients, as confirmed by postoperative CT scans (**Figure 2**).

### ASIA sensory scores

**Figure 3:**
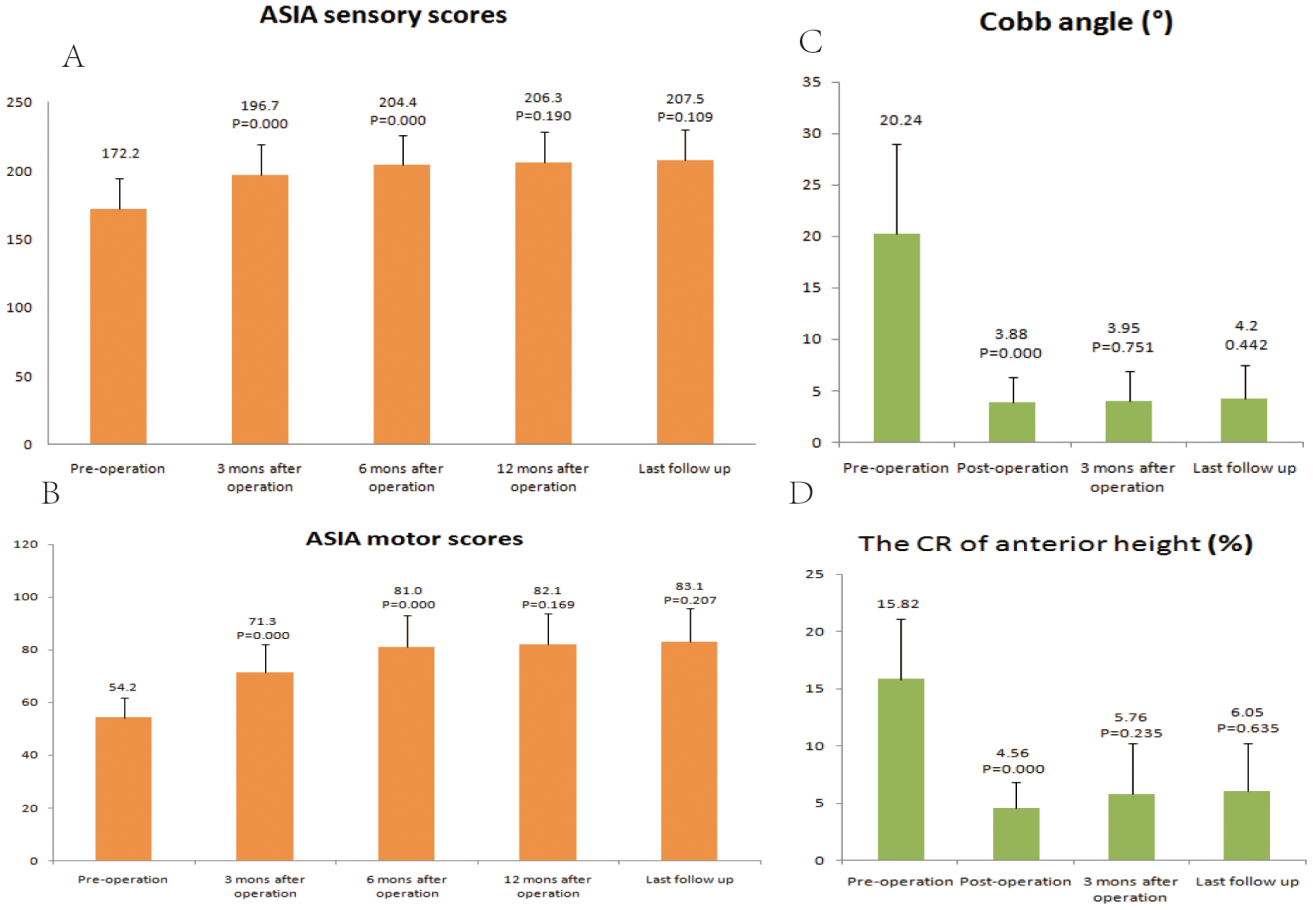
The bar graphs show the ASIA sensory scores (A), ASIA motor scores (B), Cobb angle (C), and the anterior height of two adjacent vertebrae (D) at different follow-up time points. The P values indicate the statistical difference of each follow-up time point value compared to the previous time point value. (Note: mons  =  months).

The ASIA sensory score (mean ± standard deviation) improved from 172.2±22.4 at pre-operation to 196.7±22.8 at 3 months after operation (P = 0.000), and it continued to improve to 204.4±21.7 at 6 months after operation; the improvement showed a significant difference between the 3-month and 6-month time points (P = 0.000). Slight improvements were recorded at 12 months after -operation and the last follow-up; however, no significant differences were observed when compared to the previous time points, P = 0.190 and P = 0.109, respectively (**Figure 3A**).

### ASIA motor scores

The ASIA motor score improved from 54.2±7.7 at preoperation to 71.3±10.8 at 3 months after operation (P = 0.000), and the score was 81.0±12.0 at 6 months after operation (compared to the 3-month time point, P = 0.000). Slight improvements were recorded at 12 months after operation and the last follow-up; however, no significant differences were observed when compared to the previous time points, P = 0.169 and P = 0.207, respectively (**Figure 3B**).

### Image measurement

The Cobb angle and CR of the anterior height of two adjacent vertebrae were corrected from 20.24±8.71° and 15.82±5.29% at pre-operation to 3.88±2.47° and 4.56±2.23% at postoperation, respectively (P = 0.000). The angle and CR of the anterior height were sustained at 3 months after operation (3.95±2.29° and 5.76±4.43 mm, respectively) and the last follow-up (4.20±3.23° and 6.05±4.16%, respectively), and no significant difference was found among the time points of postoperation, 3 months after operation, and the last follow-up (**Figure 3C–D**).

## Discussion

Thoracolumbar fractures and dislocation are caused by high-energy trauma such as falls or vehicular accidents, and the spinal cord is injured directly by an anterior damaged intervertebral disc and bony fragments. It has been reported that simultaneous combined anterior and posterior surgery can achieve sufficient decompression, reduction, and reconstruction [1], [13]. However, the anterior approaches have high risks due to potential anterior vascular and nerve injury [4], [14], [15].

Schmid *et al*. [16] have reported posterior lumbar interbody fusion (PLIF) in the treatment of thoracolumbar trauma and have suggested that PLIF avoids the potential complications of anterior decompression. However, the risk of damage to the conus medullaris and cauda equine due to the need for retraction limit PLIF to L3–S1 levels [7].

Harms *et al*. have developed a TLIF technique to treat degenerative disease of the lumbar spine. In addition, Humphreys *et al*. [7] have compared the techniques of TLIF and PLIF. They found that the TLIF technique has a similar operative time, duration of hospital stay, and blood loss as PLIF; has fewer complications than the PLIF procedure; maintains normal muscular attachments; and thus causes no disruption to the loading mechanics of the spine. Moreover, TLIF is not limited to L3–S1 levels.

Huang *et al*. [17] practiced the technique of transforaminal decompression and interbody fusion on a 24-year-old patient with a T11–12 Chance fracture. Similarly, Fang *et al*. [9] applied transforaminal decompression and interbody fusion to treat a 26-year-old man with an old T12–L1 fracture and dislocation. These two cases both achieved a good clinical outcome. Furthermore, Wang *et al*. [10] have reported a series of type A3 (AO classification) thoracolumbar/lumbar fractures treated by transforaminal decompression and interbody fusion; they suggest that posterior short segment pedicle screw fixation and TLIF might be an optimal surgical treatment option for A3 burst fractures and Denis type A, B, C, and E pedicle fractures.

In this study, all patients with a Type C thoracolumbar fracture and dislocation (AOSpine Thoracolumbar Spine Injury Classification System [11]) had damage mainly at the intervertebral disc, including the adjacent endplate and some part of the vertebrae. The aim of surgery is to decompress the spinal canal, reduce dislocation, and rebuild spinal stability. The transforaminal decompression and interbody fusion technique itself allows easy detection and removal of the anterior damaged intervertebral disc and bony fragments, avoiding excessive traction of the spinal cord and nerve root, thus significantly reducing the risk of nerve damage.

The follow-up data showed significant improvement of nerve function; the sensory scores and motor scores improved by 31.2±14.4 and 26.8±9.7, respectively, at 6 months after operation, and by 35.3±16.1 and 28.9±9.6, respectively, at the last follow-up. The Cobb angle was corrected by 16.36±7.98°, the CR of the anterior height of two adjacent vertebrae was restored by 11.26±4.96% at postoperation, and the correction was maintained at 3 months postoperation and the last follow-up, without internal instrument breakage or failure. Therefore, we suggest that transforaminal decompression and interbody fusion is an efficient and safe procedure for thoracolumbar fracture and dislocation. Since the technique applied in this study was not limited to the lumbar levels, we named it as “transforaminal decompression and interbody fusion,” deleting the term “lumbar” in the previous name of “transforaminal lumbar interbody fusion (TLIF).”

The limitations of our study included the inherent shortcomings of a retrospective study itself and the small sample size. Only 24 patients with at least 2-year follow-up were included because of the small proportion of thoracolumbar fracture and dislocation in thoracolumbar injuries. Moreover, the indication of this study was limited to patients in whom the damage was mainly focused on the intervertebral disc and the adjacent endplate.

## Conclusions

Based on the results of this study, we suggest that transforaminal decompression and interbody fusion is an alternative method to treat thoracolumbar fracture and dislocation when the damage is mainly at the intervertebral disk and the adjacent endplate.
